# Gender differences in melanoma awareness, diagnosis and treatment: Patient‐reported data from a multicentre survey in Switzerland

**DOI:** 10.1002/ski2.442

**Published:** 2024-09-09

**Authors:** Johanna Mangana, Cristina Lamos, Berna C. Özdemir, Heinz Läubli, Linda Morgan, Lara V. Maul, David König, Florentia Dimitriou, Sandra Kaiser, Janine Landolt, Anastasia Musiari, Nadine Pasche, Beat Siegenthaler, Reinhard Dummer, Valerio Del Prete

**Affiliations:** ^1^ Department of Dermatology University Hospital Zurich Zurich Switzerland; ^2^ University of Zurich UZH Zurich Switzerland; ^3^ Department of Dermatology HFR Fribourg – Cantonal Hospital Villars‐sur‐Glâne Switzerland; ^4^ Department of Oncology University Hospital Bern Bern Switzerland; ^5^ Division of Medical Oncology University Hospital Basel Basel Switzerland; ^6^ Department of Dermatology University Hospital of Basel Basel Switzerland; ^7^ Novartis Pharma Schweiz AG Risch‐Rotkreuz Switzerland

## Abstract

There is a need to understand the journey of patients with melanoma, including any associated gender differences, to identify aspects in the patient journey that could be improved. We used data collected from an online survey completed by adults with stage III or IV melanoma at three Swiss melanoma centres to understand patients' perceptions of their healthcare journeys from melanoma diagnosis to treatment and the relevance of treatment attributes (Figure 1). We identified that there is a need to improve communication about the testing process and gender differences in terms on information needs, relevance of treatment attributes and attitudes towards psychological support.

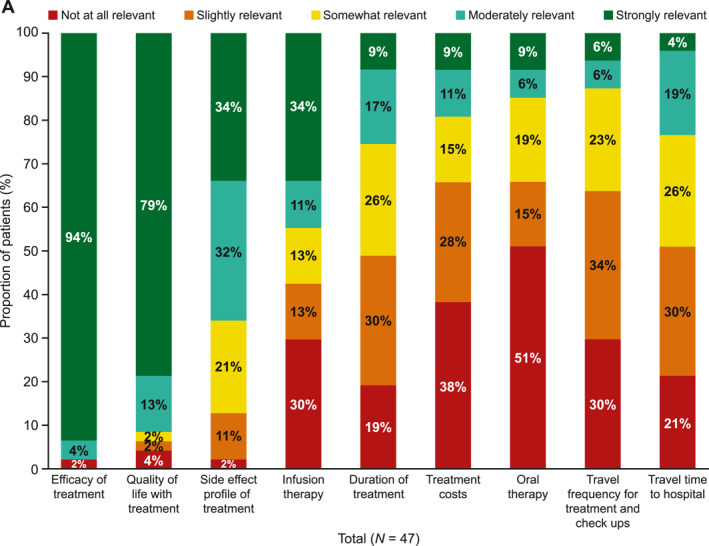

Dear Editor,

Switzerland has the seventh highest overall rate of melanoma worldwide.^1^ The most common cause of melanoma is UV radiation, associated with approximately 92% of melanomas in Switzerland in 2012.^2^ Melanoma is typically identified by the appearance of a new atypical mole or change in an existing mole and an early diagnosis significantly improves patient outcomes. Patients with stage III–IV disease typically receive systemic therapy such as immunotherapy and/or targeted therapy. Patients with BRAF V600 mutations, which occur in approximately 40%–50% of patients with advanced melanomas, may receive targeted therapy with BRAF/MEK inhibitors.^3^ It is important to understand patients' preferred treatment attributes and ensure that patients are well‐informed for decision‐making with regard to their treatment. It is also essential that patients receive support to help them cope with their diagnosis, which can improve their quality of life and outcome.^4^

Sex and gender differences in melanoma risk and outcomes are well‐documented. Male patients are approximately 1.5 times more likely to develop melanoma than female patients.^5^ Despite this, men are less likely than women to examine their skin or seek physician examinations for melanoma.^6^ Therefore, there is a need to understand differences between genders in order to provide personalised care.

The MELPAVY study used retrospective survey data collected from 47 adults (over 18 years old) with stage III–IV melanoma from three Swiss melanoma centres over a 9‐month period from March 2021 to November 2021. All patients had received a diagnosis of melanoma prior to participation in the survey. The study aim was to understand patients' perceptions of their healthcare journeys from melanoma diagnosis to treatment. Patients were also stratified by gender to identify any differences between men and women in how they receive and perceive care.

Patients were asked by their healthcare team to participate in the online survey and if they agreed, given the access details so that they could answer the questions at home. Participants were required to complete a consent form and confirm that they were aged 18 years or older and had melanoma before being able to view the questionnaire. The survey was developed in collaboration with physicians and nurses with questions covering various patient experiences, including perception of their initial diagnosis, treatment, physician care and follow‐up, the sources of information they used and the support they received. Data were collected anonymously via an online survey; therefore, follow‐up was not possible. No statistical analyses were conducted; all results presented are descriptive. The patient survey can be found in the Supplementary Materials.

Overall, 30 patients were men (64%), 25 patients had stage IV melanoma (53%) and 22 (47%) had stage III melanoma. Most men (18/30, 60%) had stage IV disease, whereas most women (10/17, 59%) had stage III disease (Supplementary Table S1). Approximately, one‐fifth of patients (17%) did not know about the disease ‘melanoma’ before being diagnosed. Of the 39 patients who knew about the disease ‘melanoma’, 37/39 (95%) knew that it can be caused by UV exposure. Most patients knew melanoma risk can be decreased by using sunscreen (33/39, 85%) and by avoiding sun exposure (30/39, 77%). Only 18/39 (46%) knew tanning beds were not safer than sitting in the sun and 14/39 (36%) knew how to self‐assess moles. These results indicate a need for improvement in melanoma prevention and increased public awareness.

National Institute for Health and Care Excellence guidance recommends that patients with melanoma are provided with accurate and easy‐to‐understand written and spoken information in a sensitive and timely manner throughout their care, tailored to their needs and circumstances.^7^ Patients were generally extremely satisfied or satisfied (24/47; 51%) or neutral (15/47; 32%) with the diagnosis communication by their treating physician. However, 8/47 (17%) of patients were extremely dissatisfied or dissatisfied. Of these, 4/8 (50%) would have liked more information on what the future/patient journey would look like and 2/8 (25%) would have liked a face‐to‐face interaction. Most men and women found that the information discussed after diagnosis is useful (19/30 [63%] and 10/17 [58%], respectively) but more women reported it ‘not useful’ or ‘not at all useful’ compared with men (5/17 [30%] vs. 2/30 [6%], respectively; Supplementary Figure S1). After diagnosis, the physician was both the preferred information source (36/47 [77%]) and the most used information source (33/47 [70%]). However, fewer women than men used information from physicians (8/17 [47%] vs. 23/30 [77%]), online cancer patient forums (1/17 [6%] vs. 3/30 [23%]) and family (1/17 [6%] vs. 6/30 [20%]), and fewer men than women used patient brochures (16/47 [53%] vs. 11/30 [65%]).

Of the patients who received treatment for melanoma (22/47, 47%), most felt involved in their treatment decision (64% strongly involved, 23% moderately involved, 5% somewhat involved and 5% slightly involved; Supplementary Figure S2). When asked about factors that influenced their decision about treatment, efficacy and quality of life were the most important factors, followed by risk of side effects (Figure 1). When asked how relevant the following attributes of melanoma treatment choices are for them, a higher proportion of women considered the side‐effect profile of treatment moderately or extremely relevant when choosing treatments compared with men (77% vs. 60%). The main concerns regarding the side effects of treatments were the possibility of long‐lasting side effects, being restricted in activities because of possible side effects, the specific side‐effect profile and the loss of quality of life (Figure 1). When asked what challenges patients with stage III melanoma had faced adhering to their current treatment, the most common answers were concerns about side effects (60%), occurrence of side effects (55%) and costs/reimbursement by health insurance (20%; Supplementary Table S2). To provide more personalised care, physicians should consider discussing side‐effect profiles of therapies in more detail when making treatment decisions with women compared with men.

**FIGURE 1:**
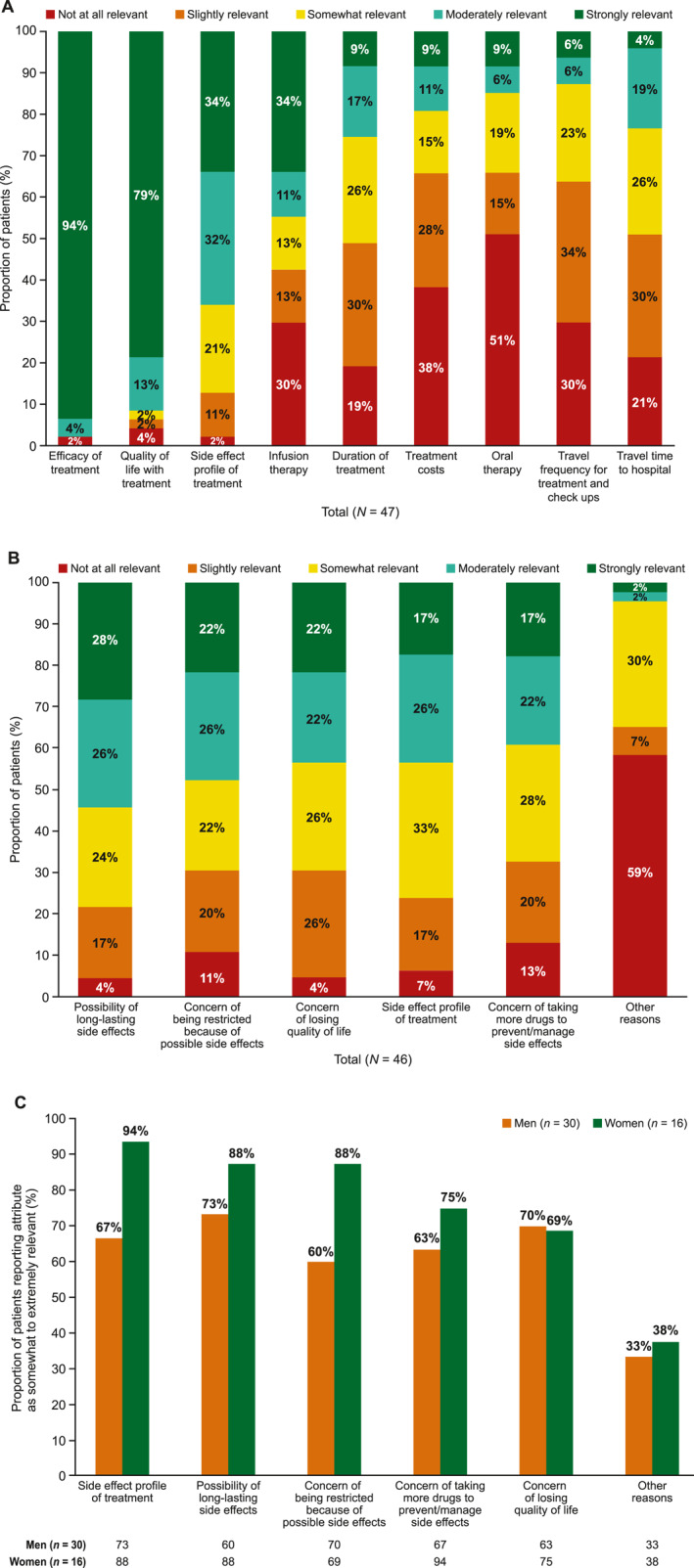
Relevance of treatment attributes of melanoma treatment. (a) Relevance of reasons of concern regarding the side‐effect profile of melanoma treatment in (b) all patients and (c) men versus women. All 47 patients were asked the following question: ‘How relevant are the following attributes of reasons of concerns regarding the side‐effect profile of the melanoma treatment for you?’. One response from patient (woman) was missing. The bars representing infusion therapy, duration of treatment and treatment costs exceed 100% owing to rounding (a). Note: The bar for ‘concern of being restricted because of possible side effects’ in 4A exceeds 100% owing to rounding (b).

*BRAF* mutation analysis is recommended for patients with stage III–IV melanoma according to the guidelines because it influences whether patients can receive BRAF‐targeted treatment.^8^ Despite this, none of the 47 patients remembered being provided with information regarding mutational testing at diagnosis. Out of all patients, most (31/47 [66%]) did not remember whether their melanoma was assessed for *BRAF* mutations. Of the 16 patients who did remember, 81% reported that their melanoma harboured a *BRAF* mutation. Of the 22 patients with stage III disease, only 4 patients (18%) remembered that they were tested for *BRAF* mutations and of these, 3 were positive for *BRAF* mutations. At the time of the survey, only 9% of patients with stage III melanoma were treated with targeted therapy (BRAF/MEK inhibitors); 91% were receiving immune checkpoint inhibitors (ICIs; e.g., programed cell death protein‐1 [PD‐1] inhibitors; note, immune therapy is not mutually exclusive to targeted therapy). This highlights a need to improve communication about the testing process and its results as well as the subjective preferences of treating teams towards ICIs. Patients were also asked about treatments they had received at any point following diagnosis. Similar to the initial treatment, nearly all patients (98%) had received immunotherapy, followed by surgery (74%), targeted therapy (28%) and radiation therapy (26%; Supplementary Table S3). Data on the order of treatments received were not collected.

In total, 92% of patients were not receiving ongoing professional psychological support, with 81% reporting that they ‘did not see the need’. Other reasons included financial concerns and being uncomfortable talking with someone else (both 2%). A higher proportion of men reported that they ‘did not see the need’ compared with women (93% vs. 63%, respectively). All four patients receiving psychological support either strongly agreed or agreed it was helpful. These findings indicate that training may help oncologists to communicate the benefits of psychological support especially to men.

A key strength of the study was that the survey was developed with support from melanoma experts and a melanoma nurse practitioner, which ensured the aspects of the patient journey that mattered most to patients were captured. However, the small sample size (*n* = 47) and lack of statistical testing may limit the conclusions that can be drawn. The study was reliant on patients' memories, so it may be subject to potential recall bias and older patients may have declined participation because of difficulties accessing the online survey.

This study provided valuable information on patients' perceptions of their healthcare journeys from diagnosis to treatment. Approximately one‐fifth of patients were not aware of the disease ‘melanoma’ before being diagnosed and less than half of patients knew how to minimise the risk and self‐assess for melanoma, which indicates a need to increase public awareness of melanoma. None of the patients included in this survey remembered being provided with information regarding BRAF mutational testing at diagnosis; considering these results heavily influence treatment eligibility, this highlights a need to improve the communication about the testing process and its results. Gender differences were found, including preferences regarding information sources and the most important treatment features when choosing a treatment, highlighting the importance of tailored patient communication to ensure patients are well‐informed for decision‐making.

## CONFLICT OF INTEREST STATEMENT

CL declared receiving institutional honoraria for lectures and advisory boards from Bristol Myers Squibb (BMS), MSD, Novartis and Sanofi. BCO declared receiving institutional honoraria for lectures and advisory boards from BMS, Ipsen, Janssen, MSD, Merck, Novartis, Pfizer, Roche and Sanofi. HL received travel grants and consultant fees from BMS, Alector, InterVenn, GlycoEra and Merck, Sharp and Dohme (MSD) and received research support from BMS, Novartis, GlycoEra and Palleon Pharmaceuticals. JM has intermittent project focused consultant or advisory relationships with Merck/Pfizer, Merck Sharp and Dohme, Amgen, Novartis, Roche, BMS and Pierre Fabre and has received travel support from Ultrasun, L’Oreal, Merck Sharp and Dohme, BMS and Pierre Fabre outside of the submitted work. VDP declared current or previous collaboration projects with Novartis, MSD, and Pierre Fabre. LM declared project focused advisory relationship with BMS, Merck, MSD and Novartis. LVM served as adviser for, and/or received speaking fees from, and/or participated in clinical trials sponsored by Almirall, Amgen, Eli Lilly, MSD, Novartis, Pierre Fabre, Roche and Sanofi (all fees to institution). DK declared honoraria (Amgen and Sanofi), attending meetings/travel (Amgen and Sanofi) and advisory boards (AstraZeneca, Merck and MSD) (all fees to institution). FD receives/received honoraria and travel support from BMS, MSD and Sun Pharma. RD declared collaboration with Novartis, MSD, BMS, Roche, Amgen, Takeda, Pierre Fabre, Sun Pharma, Sanofi, CatalYm, Second Genome, Regeneron, Alligator, T3 Pharma, MaxiVAX SA, Pfizer and touchIME outside the submitted work. SK, JL, AM, NP and BS are employees of Novartis Pharma Schweiz AG, Switzerland.

## AUTHOR CONTRIBUTIONS

**Johanna Mangana**: Conceptualization (lead); methodology (lead); writing – review & editing (lead). **Cristina Lamos**: Conceptualization (lead); methodology (lead); writing – review & editing (lead). **Berna C. Özdemir**: Conceptualization (lead); methodology (lead); writing – review & editing (lead). **Heinz Läubli**: Conceptualization (supporting); methodology (supporting); writing – review & editing (supporting). **Linda Morgan**: Methodology (supporting); writing – review & editing (supporting). **Lara V. Maul**: Conceptualization (supporting); methodology (supporting); writing – review & editing (supporting). **David König**: Conceptualization (supporting); methodology (supporting); writing – review & editing (supporting). **Florentia Dimitriou**: Conceptualization (supporting); methodology (supporting); writing – review & editing (supporting). **Sandra Kaiser**: Conceptualization (supporting); methodology (supporting); writing – review & editing (supporting). **Janine Landolt**: Conceptualization (supporting); formal analysis (supporting); methodology (supporting); writing – review & editing (supporting). **Anastasia Musiari**: Methodology (supporting); writing – review & editing (supporting). **Nadine Pasche**: Methodology (supporting); writing – review & editing (supporting). **Beat Siegenthaler**: Methodology (supporting); writing – review & editing (supporting). **Reinhard Dummer**: Conceptualization (supporting); methodology (supporting); writing – review & editing (supporting). **Valerio Del Prete**: Conceptualization (supporting); methodology (supporting); writing – review & editing (supporting).

## FUNDING INFORMATION

This study was funded by Novartis Pharma Schweiz AG, Switzerland.

## ETHICS STATEMENT

Not applicable.

## PATIENT CONSENT

Not applicable.

## Supporting information

Supporting Information S1

Supporting Information S2

## Data Availability

Data available on request. The data underlying this article will be shared on reasonable request to the corresponding author.
