# Supplementary material for: Gender differences in melanoma awareness, diagnosis and treatment: Patient‐reported data from a multicentre survey in Switzerland
Source: Skin Health Dis. 2024 Sep 9;4(6):e442. doi: 10.1002/ski2.442 (PMC11608883; doi:10.1002/ski2.442)
Supplement: Supplementary file 1 — Supporting Information S1 [file SKI2-4-e442-s002.docx]

**Gender differences in melanoma awareness, diagnosis, and treatment: patient-reported data from a multicentre survey in Switzerland**

## Supplementary Materials

SUPPLEMENTARY TABLE 1 Patient characteristics.

| **Characteristic** | **Gender** | | | | **Total**  **(*N* = 47)** |
| --- | --- | --- | --- | --- | --- |
|  | **Men (*n* = 30)** | | **Women (*n* = 17)** | |  |
| **Stage of melanoma** | | | | | |
| Stage III (%) | 12 (54.5) | 10 (45.5) | | 22 (46.8) | |
| Stage IV (%) | 18 (72.0) | 7 (28.0) | | 25 (53.2) | |
| **Age** | | | | | |
| Mean age, years (SD) | 61.5 (11.8) | 55.1 (13.6) | | 59.2 (12.8) | |
| Age range, years, n (%) | | | | | |
| 30–39 | 1 (3.3) | 4 (23.5) | | 5 (10.6) | |
| 40–49 | 4 (13.3) | 2 (11.8) | | 6 (12.8) | |
| 50–59 | 7 (23.3) | 2 (11.8) | | 9 (19.1) | |
| 60–69 | 11 (36.7) | 7 (41.2) | | 18 (38.3) | |
| 70–79 | 6 (20.0) | 2 (11.8) | | 8 (17.0) | |
| 80–89 | 1 (3.3) | 0 | | 1 (2.1) | |

SD, standard deviation.

SUPPLEMENTARY TABLE 2 Challenges of current treatment for patients with stage III melanoma.

| **Response** | **Proportion of patients, n (%)**  **(*n =* 20)** |
| --- | --- |
| Worry about side effects | 12 (60) |
| Occurrence of side effects | 11 (55) |
| Costs/reimbursement by my health insurance | 4 (20) |
| Too many medications | 2 (10) |
| Depression | 2 (10) |
| Want to have a break | 1 (5) |
| Restriction on daily life due to fasting requirement | 1 (5) |
| Restrictions on daily life due to travel to treatment site | 1 (5) |
| Other | 3 (15) |
| Forgot specific challenges that I faced | 1 (5) |
| None | 4 (20) |

SUPPLEMENTARY TABLE 3 Treatment received at the time of the survey and during the entire disease period.

| **Response** | **Proportion of patients, n (%)** |
| --- | --- |
| Treatment received at time of survey (patients with stage III melanoma only) | ***n* = 22** |
| Immunotherapy infusions (e.g. PD-1 inhibitor)^a^ | 20 (91) |
| Targeted therapy,^a^ tablets (e.g. BRAF/MEK inhibitors) | 2 (9) |
| Treatment received during entire disease period | ***n* = 47** |
| Immunotherapy infusions (e.g. PD-1 inhibitor) | 46 (98) |
| Surgery | 35 (74) |
| Targeted therapy, tablets (e.g. BRAF/MEK inhibitors) | 13 (28) |
| Radiation therapy | 12 (26) |
| Investigational clinical trial | 8 (17) |
| Chemotherapy (infusion, tablets) | 7 (15) |
| Alternative/holistic treatments | 4 (9) |

BRAF, V-Raf Murine Sarcoma Viral Oncogene Homolog B; MEK, mitogen-activated protein kinase kinase; PD-1, programmed cell death protein 1.

^a^Immune therapy is not mutually exclusive to targeted therapy.

SUPPLEMENTARY FIGURE 1 (A) Information provided at first diagnosis by healthcare professional and (B) information sources used following diagnosis stratified by gender.


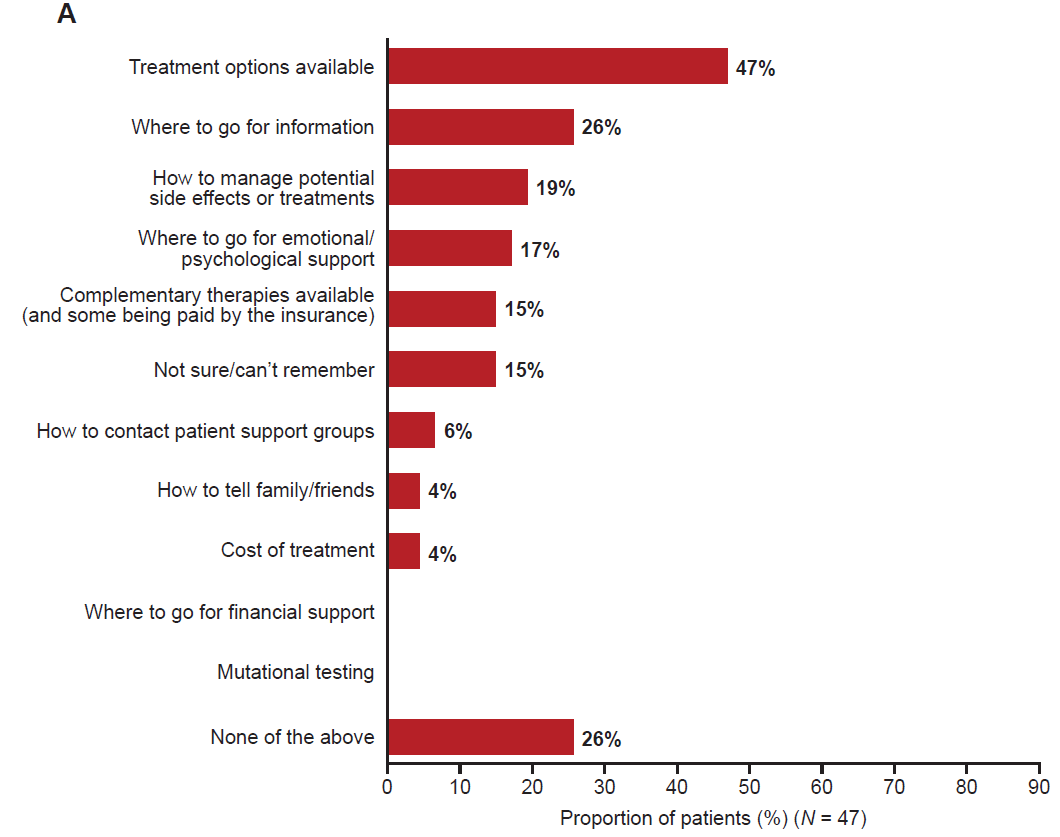


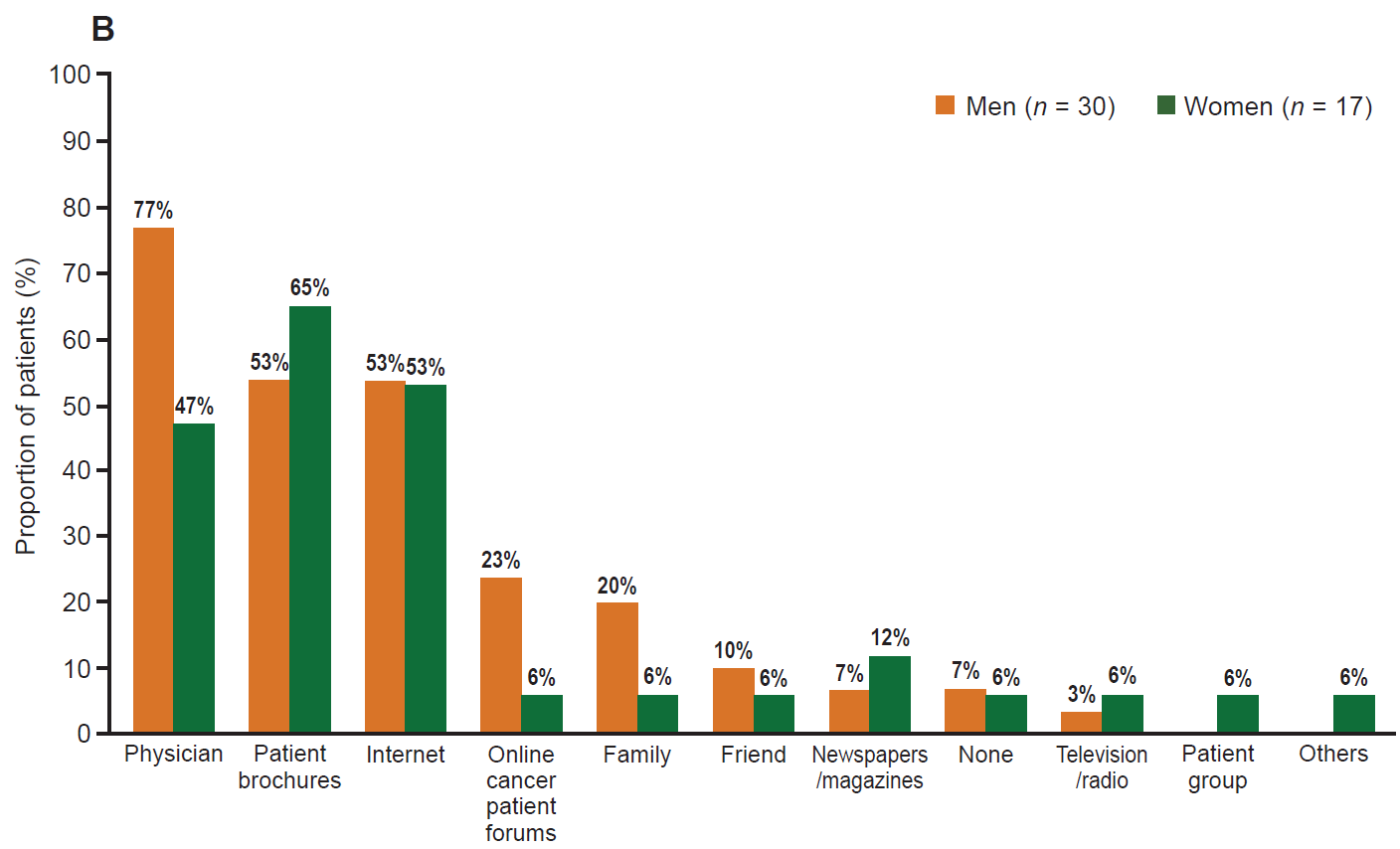


(A) All 47 patients were asked the following question: “At the time of your diagnosis, did the healthcare professional tell you about: Patients were allowed to select multiple responses. (B) All 47 patients were asked the following question: “What information sources for melanoma patients did you use to gain knowledge on melanoma – just after you were diagnoses with melanoma”. Patients were allowed to select multiple responses.

SUPPLEMENTARY FIGURE 2 Involvement in the treatment decision.


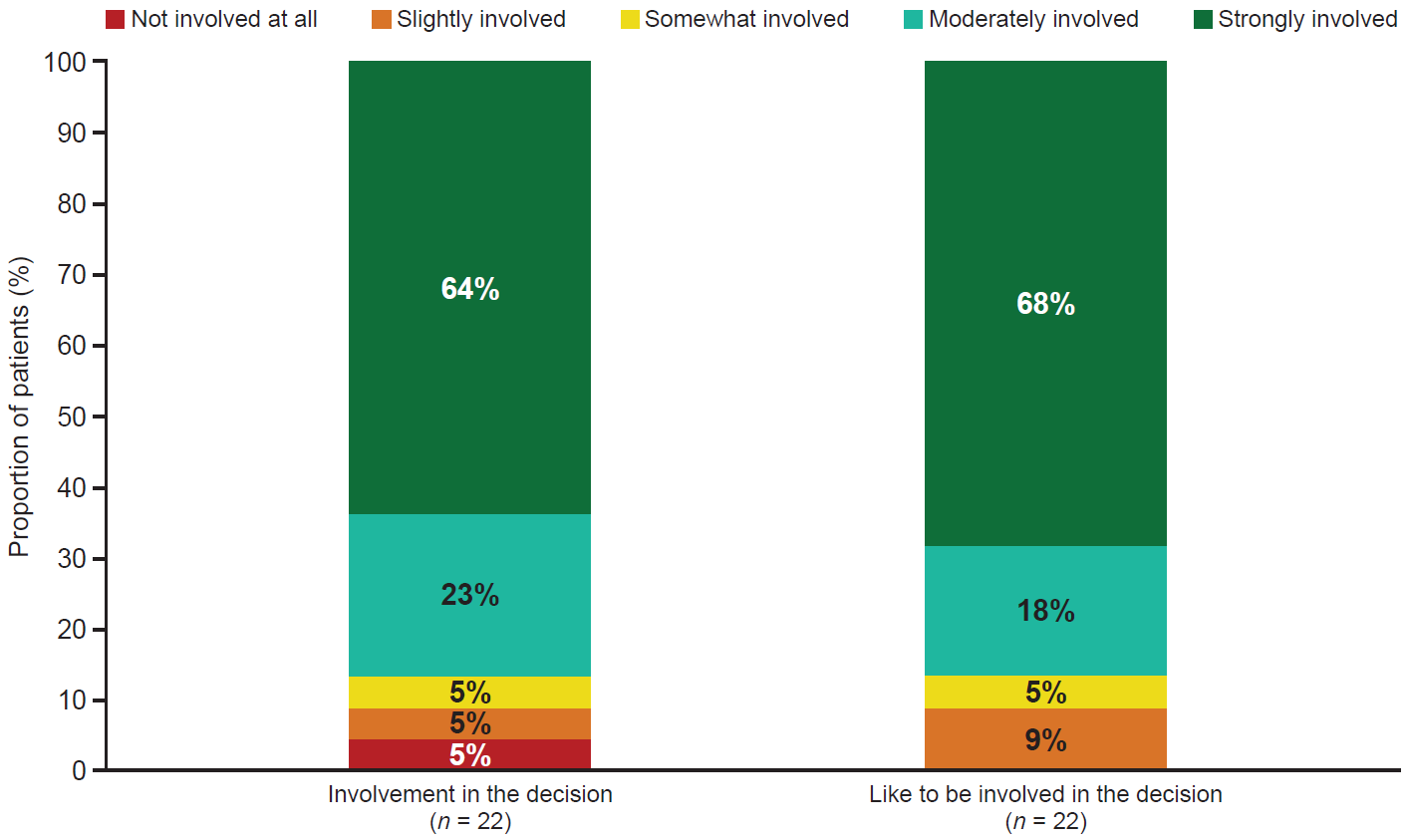


Patients who received treatment for melanoma (22/47) were asked the following questions: “How strongly were you involved in the decision for your treatment?” and “How strongly would you have liked to be involved in the decision for your treatment?”.

Note: the sum of the left-hand bar exceeds 100% (102%) owing to rounding.
