# Supplementary material for: Gender differences in melanoma awareness, diagnosis and treatment: Patient‐reported data from a multicentre survey in Switzerland
Source: Skin Health Dis. 2024 Sep 9;4(6):e442. doi: 10.1002/ski2.442 (PMC11608883; doi:10.1002/ski2.442)
Supplement: Supplementary file 2 — Supporting Information S2 [file SKI2-4-e442-s001.pdf]

MELPAVEY – a quantitative melanoma patient survey to design a better melanoma patient experience

Contents

1. **LANGUAGE** 2
2. **LOG IN** 3
3. **SCREENER** 4
   INFORMATION AND AGREEMENT 4
   PATIENT SCREENER 6
4. **MAIN** 8
   SECTION S: STAGING 8
   SECTION I: INVOLVEMENT 10
   SECTION T: TREATMENT START 12
   SECTION C: CURRENT TREATMENT STATUS 14
   SECTION D : MELANOMA AWARENESS , DIAGNOSIS & REFERRAL 30
5. **END** 37

1. LANGUAGE

Please select your preferred language.

○	Deutsch / German / Allemand
○	Français / French / Französisch
○	English / Englisch / Anglais
○	Nederlands / Dutch / Néerlandais

Logic	Start German version if 1; Start French version if 2

2. LOG IN

Please log in with your personal password.

○	PASSWORD

Logic	Verify password

3. SCREENER

INFORMATION AND AGREEMENT

Thank you for agreeing to participate in this survey on the subject of melanoma, more specifically stage III and stage IV melanoma.
The overall aim is to design together with doctors and nurses an even better patient experience.

The survey covers different areas, such as patient experiences (from initial diagnosis, treatment(s), to physician care and follow-up), available sources of information and support. From the results, we hope to identify starting points for future targeted support for melanoma patients.

Pharmacovigilance:

If you raise an adverse event related to a marketed drug in the course of this market research, we are required to inform the drug safety department of the manufacturer. When reporting the adverse event, your personal information will remain anonymous. In some cases, the manufacturer may need additional information from you to properly classify the adverse event. If the drug safety department needs more information about the event: Are you willing to give up the confidentiality imposed on us under the Code of Conduct for Market Research Studies with respect to this event so that you can be contacted for additional information?

If you provide your name as part of an adverse event report, it will be linked exclusively to that report and shared only with the Drug Safety Department. Your name will NOT be linked IN ANY WAY to the other responses you provide as part of this market research. Anything you tell us during the survey will continue to be kept confidential.

*Your response will NOT affect your participation in this survey.

1	❍	Yes, I agree - exclusively in connection with an adverse event - to the disclosure of my name and contact details.
2	❍	No, I do not consent to the disclosure of my name and contact information in connection with an adverse event. I would like to remain anonymous.

Data privacy:

- The protection of your data is guaranteed.
- Your personal answers remain anonymous and will not be passed on to your doctor or other persons.
- Your assistance by participating in this survey does not change your treatment, nor does it affect your relationship with your doctor or the quality of your care.

By entering the survey link, you understand and agree to the following:

- you allow the market research agency (Polyquest) to share the summarized results of the survey (but not your personal answers) with Novartis, the participating oncologists and patient representatives, and with persons or companies that work with Novartis in this context.
- you allow the results to be published, still under full anonymization.

This survey is initatiated and sponosored by Novartis Pharma Schweiz AG and conducted by Polyquest AG.

I confirm that I have read and understood the above information. I am aware that I do not have to participate and that I can stop filling out the questionnaire at any time.

If you wish to participate, please give your consent here by answering "Agree".

○	Agree
○	Disagree -> KEIN INTERVIEW

Logic	Screen out if code 2 ‘disagree’
Button	The following text only appears if the respondent clicks the button.

PATIENT SCREENER

ASK ALL

S1.

Were you ever diagnosed with melanoma?
Please select 1 option
Single code only

1	Yes	❍		
2	No	❍		➔terminate

ASK ALL

S2.

How old are you?

yyyy

Logic	If<18 -> SCREENOUT

ASK ALL

S3.

Please select your gender:
Please select 1 option
Single code only

	Gender	
1	Male	❍
2	Female	❍
3	Other	❍

ASK ALL

S4a.

Which of the following countries do you primarily live in?
Please select 1 option
Single code only

	Countries		
1	Germany	❍	➔country-specific Q're if availble, otherwise terminate
2	France	❍	➔country-specific Q're if availble, otherwise terminate

3	Italy	❍	→terminate
4	Switzerland	❍	
5	Austria	❍	→country-specific Q're if availble, otherwise terminate
6	Netherlands	❍	→country-specific Q're if availble, otherwise terminate
7	Belgium	❍	→country-specific Q're if availble, otherwise terminate
8	Ireland	❍	→country-specific Q're if availble, otherwise terminate
9	Luxemburg	❍	
6	Other	❍	→terminate

4. MAIN

SECTION S: STAGING

ASK ALL

S6a.

What melanoma stage were you diagnosed most recently? (please fill the answer provided by your physician or skin cancer nurse on a separate paper)
Please select 1 option
Single code only
Provide a link to melanoma stage definition

	melanoma stages		
1	Stage 0 melanoma	❍	→terminate
2	Stage 1 melanoma	❍	→terminate
3	Stage 2 melanoma	❍	→terminate
4	Stage 3 melanoma	❍	
5	(expand if S6a=4) Stage 3a melanoma	❍	
6	(expand if S6a=4) Stage 3b melanoma	❍	
7	(expand if S6a=4) Stage 3c melanoma	❍	
8	(expand if S6a=4) Stage 3d melanoma	❍	
9	Stage 4 melanoma	❍	

ASK ALL

S6b.

When where you diagnosed with **[PN answer of S6a]**? (please fill the answer provided by your physician or skin cancer nurse on a separate paper)

	MONTH **[RANGE (January till December)]**
	YEAR **[RANGE (2000,2021)]**
Logic	If YEAR<2000 -> SCREENOUT

ASK ALL

S6a1.

What stage of melanoma did you have at the initial diagnosis? (please fill the answer provided by your physician or skin cancer nurse on a separate paper)
Please select 1 option
Single code only

Provide a link to melanoma stage definition

	melanoma stages		
1	Stage 0 melanoma	❍	→terminate
2	Stage 1 melanoma	❍	→terminate
3	Stage 2 melanoma	❍	→terminate
4	Stage 3 melanoma	❍	
5	(expand if S6a=4) Stage 3a melanoma	❍	
6	(expand if S6a=4) Stage 3b melanoma	❍	
7	(expand if S6a=4) Stage 3c melanoma	❍	
8	(expand if S6a=4) Stage 3d melanoma	❍	
9	Stage 4 melanoma	❍	
10	I don't know	❍	

ASK ALL

S6b1.

When where you initially diagnosed with [PN answer of S6a1]? (please fill the answer provided by your physician or skin cancer nurse on a separate paper)

	MONTH [RANGE (January till December)]
	YEAR [RANGE (2000,2021)]
Logic	If YEAR<2000 -> SCREENOUT

SECTION I: INVOLVEMENT

ASK ALL

I0.

Were you referred from a peripheral hospital to a specialized center/university hospital?

Please select 1 option

Single code only

1	Yes	❍
2	No	❍

ASK ALL

I1.

Did you get a 2nd opinion on the most recent diagnosis?

Please select 1 option

Single code only

1	Yes	❍
2	No	❍

ASK if I1=2

I2.

Why did you not get a 2nd opinion on this diagnosis?

	Reasons for not getting a 2nd opinion	
1	Too expensive	❍
2	Too time consuming	❍
3	I was not expecting further benefit from input from another doctor	❍
4	Other reason(s)	❍

ASK ALL

I3a.

Did you receive a pathology report?

	Reasons for not getting a 2nd opinion	
1	Yes	❍
2	No	❍
3	I don't know	❍

ASK if I3a=1

I3b.

Was the pathology report explained to you?

1	Yes	❍
2	No	❍
3	I don't know	❍

ASK if I3a=1

I3c.

Did the oncologist explain to you what impact a mutation on your disease has?

1	Yes	❍
2	No	❍
3	I don't know	❍

ASK all

I4.

To what extent do you agree with the statements?

		Fully agree	Rather agree	Rather not agree	Do not agree at all
1	I am well informed about my disease	○	○	○	○
2	I feel supported by my doctor	○	○	○	○
3	My doctor understands my situation	○	○	○	○

SECTION T: TREATMENT START

<u>ASK if S6a=4,5,6,7</u>

T1.

Are you receiving or have you recently received treatments for melanoma for which you received infusions or injections or had to swallow tablets (so-called **systemic therapy**)?
Please select 1 option
Single code only

1	Yes I am still receiving this kind of treatment	❍
2	Yes I received this kind of treatment but currently I am not receiving such a treatment	❍
3	No, I never received such treatment	❍

ASK if T1=2,3 and S6a=4,5,6,7

T2.

Why are you not receiving / did you not receive such systemic treatment (for example tablets)?
Select all that apply
Multi code possible

	Reasons for not being under melanoma treatment	
1	Personal decision	❍
2	Physician's decision	❍
3	Financial hurdles	❍
4	Afraid of side effects	❍
5	Planned treatment period ended	❍
6	Other reason	❍

ASK if T2=1,2

T5.

Were you offered the possibility of a second opinion on your treatment choice(s)?
Select all that apply
Single code

1	Yes, I was given names and addresses	❍
2	Yes, was mentioned	❍
3	No	❍
4	No, but I would have wished	❍
5	Other	❍

[All terminations]

Thank you for taking the time to respond to our request. Unfortunately, we have already exceeded our participation quota for patients with your background. We appreciate your interest and hope that you will participate in future surveys.

Introduction
[ASK ALL]
The following questions will focus on your patient journey. Thank you in advance for your valuable input.

SECTION C: CURRENT TREATMENT STATUS

ASK ALL

C1a.

In which kind of medical facility are you currently being cared for?
Please select 1 option
Single code only

	Treatment facility	
1	Independent private practice or oncology center (not affiliated with a hospital)	❍
2	Smaller regional hospital	❍
3	Large regional hospital	❍
3	University Hospital	❍
4	Other	❍

ASK ALL

C2a.

How do you usually travel to the treatment facility?
Single code only

	Transport options to treatment facility	
1	Walking by feet	❍
2	Driving myself	❍
3	Driven by a family member/friend	❍
4	Taxi	❍
5	Public Transport	❍
6	Medical driving service	❍
7	Other	❍

ASK ALL

C2b.

How long does it take you to travel to this treatment facility from your home (one way only)?

	Time for travelling	
1	Less than 15 minutes	❍
2	15 to 30 minutes	❍
3	31 to 45 minutes	❍
4	46 to 60 minutes	❍
5	More than 1 hour to 1.5 hours	❍
6	More than 1.5 hours	❍

ASK ALL

C3a.

How satisfied are you with this treatment facility?

	Satisfaction with treatment centre	
1	Extremely Dissatisfied	❍
2		❍
3	Neutral	❍
4		❍
5	Extremely Satisfied	❍

ASK ALL

C4a.

Was your melanoma assessed for BRAF mutations?
Please select 1 option
Single code only

1	Yes	❍
2	No	❍
3	I don’t know	❍

ASK if S6a=8

C4b1.

Does your melanoma harbor a BRAF mutation?

Please select 1 option

Single code only

	BRAF mutation status	
1	Yes	❍
2	No	❍
3	I don't know	❍

ASK if C4a=1

C4b2.

How long did you wait for the results of your mutational analysis?

Please select 1 option

Single code only

	BRAF mutation status	
1	Less then 1 week	❍
2	Between 1-2 weeks	❍
3	Between 3-4 weeks	❍
4	More than 4 weeks	❍
5	I don't know	❍

ASK if C4a=1

C4c.

When during your patient journey were you tested for the BRAF mutation?

Please select 1 option

Single code only

	Mutation testing stage	
1	After removing of the mole	❍
2	After lymph node biopsy / surgery and before treatment initiation	❍
3	After disease recurrence / progression	❍
4	Other time point	❍
5	I don't know	❍

ASK if C4a=1

C4d.

Who made you aware about BRAF mutation testing for melanoma?

Select all that apply

Multi code possible

	BRAF testing awareness source	
1	Primary care physician	❍
2	(Dermato-)Oncologist	❍
3	Dermatologist	❍
4	Nurse	❍
5	My own research	❍
6	Cancer support group	❍
7	Other	❍

ASK IF T1=1,2

C5a.

You mentioned that you are being currently treated or have been treated for [Enter S6a].
How strongly were you involved / would you have liked to be involved in the decision for this treatment?

	Treatment decision involvement	Not involved at all (1)	Slightly involved (2)	Somewhat involved (3)	Moderately involved (4)	Strongly involved (5)
1	Extent to which I was involved.	❍	❍	❍	❍	❍
2	Extent to which I would have liked to be involved	❍	❍	❍	❍	❍

ASK IF T1=1,2

C5c

How long after your recent diagnosis for [Enter S6a] did you start a treatment against it?

	WEEKS<br>[RANGE (0 ,60)]
	I don't know

ASK ALL

C5d1.

What kind of treatment(s) did you receive during your entire melanoma disease period?

Select all that apply

Multi code possible

	Treatment	

1	Radiation therapy	❑
2	Targeted therapy, tablets (eg BRAF/MEK inhibitor)	❑
3	Immunotherapy, infusions (eg PD-1 Inhibitor)	❑
4	Surgery	❑
5	Investigational Clinical Trial	❑
6	Chemotherapy (infusion, tablets)	❑
7	Alternative / holistic treatments	❑
8	Other(s)	❑
9	I don't know	❑

ASK IF T1=1,2

C5d2.

How are you currently being treated or have recently been treated for [Enter S6a]?

Single code?

	Treatment	
1	Radiation therapy	❑
2	Targeted therapy, tablets (eg BRAF/MEK inhibitor)	❑
3	Immunotherapy, infusions (eg PD-1 Inhibitor)	❑
4	Surgery	❑
5	Investigational Clinical Trial	❑
6	Chemotherapy (infusion, tablets)	❑
7	Alternative / holistic treatments	❑
8	Other	❑
9	I don't know	❑

ASK IF T1=1,2

C5d3.

How long have you been treated with [mention Therapy chosen at C5d2] for your melanoma condition?

❑	MONTHS [RANGE (0 ,60)]
❑	I don't know

ASK if ASK if T1=2

C5d4.

You mentioned that you are no longer receiving [mention Therapy chosen at C5d2] for your melanoma condition.

Why did you stop treatment with [mention Therapy chosen at C5d2]?

Select all that apply

Multi code possible

	Reasons for stopping treatment	
1	Planned completion of therapy	❑
2	Due to side effects	❑

3	Due to personal decision	❍
4	Progression / Recurrence of disease	❍
5	Other reason	❍

ASK if T1=1,2

C5d5.

How often do/did you visit your (Dermato-)oncologist after starting [answer of S6a] treatment during the treatment period ?

Please select 1 option

Single code only

	Frequency of Regular visit	
1	More than twice a month	❍
2	Twice a month	❍
3	Approximately once a month	❍
4	Every 2-3 months	❍
5	Every 4-6 months	❍
6	Less than every 6 months	❍

ASK if T1=2

C5d6.

How often do you visit your (Dermato-)oncologist after the previous [answer of S6a] treatment ended?

Please select 1 option

Single code only

	Frequency of Regular visit	
1	More than twice a month	❍
2	Twice a month	❍
3	Approximately once a month	❍
4	Every 2-3 months	❍
5	Every 4-6 months	❍
6	Less than every 6 months	❍
7	No follow up visits	❍

ASK ALL

C5d7.

What do the visit(s) with your oncologist usually consist of?

Select all that apply

Multi code possible

	Follow up	
1	General discussion	❍
2	Skin checks	❍

3	Blood Tests	❍
4	Imaging (PET/CT Scan or MRI)	❍
5	Other assessments	❍
6	I don't remember	❍

ASK ALL

C5e.

How relevant are the following attributes of melanoma treatment choices for you?

	Treatment Attribute rating	Not at all relevant (1)	Slightly relevant (2)	Somewhat relevant (3)	Moderately relevant (4)	Extremely relevant (5)
1	Side effect profile of treatment	❍	❍	❍	❍	❍
2	Efficacy of treatment	❍	❍	❍	❍	❍
3	Quality of life with treatment	❍	❍	❍	❍	❍
4	Travel time to hospital	❍	❍	❍	❍	❍
5	Duration of treatment	❍	❍	❍	❍	❍
6	Travel frequency for treatment and check ups	❍	❍	❍	❍	❍
7	Treatment costs	❍	❍	❍	❍	❍
8	Oral therapy	❍	❍	❍	❍	❍
9	Infusion therapy	❍	❍	❍	❍	❍

ASK all

C5x.

How relevant are the following attributes of **oral melanoma treatment** choices for you?

	Treatment Attribute rating	Not at all relevant (1)	Slightly relevant (2)	Somewhat relevant (3)	Moderately relevant (4)	Extremely relevant (5)
1	Amount of pills per day	❍	❍	❍	❍	❍
2	Size of pills					
3	Regular daily intake of pills (ie every morning and evening)					
4	Whether intake is once or twice per day					
5	Timing of intake (eg 1h before or 2hrs after food)					
6	Food restrictions					

ASK all

C5y.

How relevant are the following attributes of **melanoma treatment administered by infusion** for your choice?

	Treatment Attribute rating	Not at all relevant (1)	Slightly relevant (2)	Somewhat relevant (3)	Moderately relevant (4)	Extremely relevant (5)
1	Infusion duration	❍	❍	❍	❍	❍
3	Frequency of infusion	❍	❍	❍	❍	❍

ASK if C5e1=2, 3, 4, 5

C5f.

You have mentioned that side effect profile of melanoma treatment is relevant for you.
How concerned are you with the side effect profiles of your current melanoma treatment?

	Level of Concern	
1	Not at all concerned	❍
2	Slightly concerned	❍
3	Somewhat concerned	❍
4	Moderately concerned	❍
5	Extremely concerned	❍

ASK if C5e1=2, 3, 4, 5

C5f1.

How relevant are the following attributes of **reasons of concerns regarding the side effect profile of the melanoma treatment** for you?

	Reason for concern	Not at all relevant (1)	Slightly relevant (2)	Somewhat relevant (3)	Moderately relevant (4)	Extremely relevant (5)
1	Concern of suffering because of side effects	❍	❍	❍	❍	❍
2	Concern of losing quality of life	❍	❍	❍	❍	❍
3	Possibility of long-lasting side effects	❍	❍	❍	❍	❍
4	Concern of being restricted because of possible side effects	❍	❍	❍	❍	❍
5	Concern of taking more drugs to prevent/ manage side effects	❍	❍	❍	❍	❍
6	Other reasons	❍	❍	❍	❍	❍

ASK if C5e=2, 3, 4, 5
Select all that apply

C5f2.

How well have you been informed in advance about the side effects of your treatment?
Please select 1 option
Single code only

	Side effect information rating	Not at all informed (1)	Slightly informed (2)	Somewhat informed (3)	Moderately informed (4)	Extremely well informed (5)
1	Informed about possible side effects of my treatment	❑	❑	❑	❑	❑

ASK IF T1=1,2

C5g.

Do/did you face any restrictions in every day life due to side effects?

Please select 1 option

Single code only

1	Yes	❑
2	No	❑

ASK IF T1=1,2

C5h.

In case you suspect or experience a side effect of your treatment, do you contact your health care professional?

	Level of contact	
1	Always	❑
2	Most often	❑
3	From time to time	❑
4	Seldom	❑
5	Never	❑

ASK IF T1=1,2 and C5h=1,2,3,4

C5i.

Which healthcare professional do you contact then?

	Level of contact	
1	Dermato-oncologist	❑
2	Nurse / Nursing team	❑
3	melanoma/Skin cancer nurse	❑
4	Primary care physician	❑
5	Emergency	❑
6	Other(s)	❑

ASK IF T1=1,2 and C5h=1,2,3,4

C5k.

How easily do you get hold of the health care professional?

	Satisfaction with treatment centre	
1	Extremely easy	❍
2		❍
3	Neutral	❍
4		❍
5	Extremely hard	❍

ASK IF T1=1 **C7b.**

What kind of challenges have you faced adhering to your current treatment regimen?

Select all that apply

Multi code possible

	Adherence / Compliance challenges	
1	None	❍
2	Worry about side effects	❍
3	Costs / Reimbursement by my health insurance	❍
4	Too many medications	❍
5	Depression	❍
6	Occurrence of side effects	❍
7	Want to have a break	❍
8	Restrictions on daily life due to fasting requirement	❍
9	Restrictions on daily life due to travel to treatment site	❍
10	Other	❍
11	Forgot specific challenge which I faced	❍

ASK ALL

C8a.

How strongly involved are the following caregivers in managing your melanoma?

Please select 1 option

Single code only

	Caregivrs	Not at all involved (1)	Slightly involved (2)	Somewhat involved (3)	Moderately involved (4)	Extremely involved (5)
1	Physician	❍	❍	❍	❍	❍
2	Sibling	❍	❍	❍	❍	❍
3	Parents	❍	❍	❍	❍	❍
4	Spouse or partner	❍	❍	❍	❍	❍
5	Son or daughter	❍	❍	❍	❍	❍
6	Another family member	❍	❍	❍	❍	❍
7	A friend	❍	❍	❍	❍	❍
8	Outpatient care (e.g. Spitex) or other service	❍	❍	❍	❍	❍
9	Other	❍	❍	❍	❍	❍

ASK all

C8b.

At what stage did your primary caregiver (excluding your physician) learn about your melanoma diagnosis?
Please select 1 option
Single code only

	Stage where caregiver informed about melanoma	
1	My caregiver was with me when I received the diagnosis from the physician	❍
2	I told my caregiver about my diagnosis before my treatment decision	❍
3	I told my caregiver about my diagnosis after my treatment decision	❍
4	Other timepoint	❍

ASK all

C8c.

How does your primary caregiver (excluding your physician) support you in your melanoma treatment?
Select all that apply
Multi code possible

	Primary caregiver support	
1	Transportation to treatment centers	❍
2	Supports me with my treatment adherence	❍
3	Discussing and choosing different treatments	❍
4	Discussing and choosing different physicians	❍
5	Discussing and choosing different treatment centers	❍
6	Psychological support	❍
7	Financial support	❍
8	Other	❍

ASK ALL

C9a1.

Are you currently receiving professional Psychological support for your melanoma condition?
Please select 1 option
Single code only

1	Yes	❍
2	No	❍

ASK if C9a1=2

C9a2.

Why are you not currently receiving Professional Psychological support for your melanoma condition?
Select all that apply
Multi code possible

	Reasons for not receiving Professional Psychological support	
1	Do not see the need	❍
2	Didn't know it exists	❍
3	Financial concerns	❍
4	Not comfortable to talk with someone else	❍
5	Other	❍

ASK if C9a1=1

C9b.

Who recommended you to receive Psychological support for your melanoma condition?
Select all that apply
Multi code possible

	Recommending Psychological support	
1	Treating Physician	❍
2	Diagnosing Physician	❍
3	Nurse	❍
4	Partner	❍
5	Family	❍
6	Friends / Colleagues	❍
7	Patient support group	❍
8	No one	❍
9	Other	❍

ASK if C9a1=1

C9c.

How frequently do you have consultations with your (psycho-)therapist for your melanoma condition?
Please select 1 option
Single code only

	Therapy consultation frequency	
1	Less than one consultation every 6 months	❍
2	A consultation every 3-6 months	❍
3	A consultation every 1-2 months	❍

4	2-4 consultations per month	❍
5	> 4 consultations per month	❍

ASK if C9a1=1

C9d.

Do you agree that the professional psychological support that you are receiving as a melanoma patient is helpful?

	Level of agreement	
1	Strongly disagree	❍
2	Disagree	❍
3	Neither disagree nor agree	❍
4	Agree	❍
5	Strongly agree	❍

ASK ALL

C10a.

Do you engage with Patient support groups for melanoma patients?

Please select 1 option

Single code only

1	Yes	❍
2	No	❍

ASK if C10a=2

C10b.

Why are you not engaging with any patient support group for your melanoma condition?

Select all that apply

Multi code possible

	Reasons for not engaging with Patient support group	
1	Do not see the need	❍
2	Didn't know it exists	❍
3	Do not want to talk about my condition	❍
4	Group location is too far away	❍

5	Language barrier	❑
6	Other reason	❑

ASK if C10a=1

C10c.

Do you agree that Patient Support groups are helpful for your melanoma condition?

	Level of agreement	
1	Strongly disagree	❑
2	Disagree	❑
3	Neither disagree nor agree	❑
4	Agree	❑
5	Strongly agree	❑

ASK ALL

C11b.

How concerned are you about funding/reimbursement for/of your melanoma treatment?

	Level of concern with source of funding	
1	Not at all concerned	❑
2	Slightly concerned	❑
3	Somewhat concerned	❑
4	Moderately concerned	❑
5	Extremely concerned	❑

ASK ALL

C12.

What Information sources do you use to gain knowledge on melanoma?
Which of these information sources do you prefer?

	information sources	Used C12a Multi code	Preferred C12b Single code
1	Physician	❑	❑
2	Nurse	❑	❑
3	Other clinical staff (such as psychological therapist)	❑	❑

4	Patient brochures	❍	❍
5	Online Cancer patient forums	❍	❍
6	Colleague	❍	❍
7	Internet	❍	❍
8	Patient group	❍	❍
9	Newspapers / Magazines	❍	❍
10	Television / Radio	❍	❍
11	Family	❍	❍
12	Other sources	❍	❍
13	None	❍	❍

ASK ALL

C12c.

On which topics would you like to have more information?

Select all that apply

Multi code possible

	More Information needed on	
1	Treatment options	❍
2	Nutrition	O
3	Disease background	❍
4	Psychological support available	❍
5	Side effect and their management	❍
6	Funding sources available	❍
7	Alternative and/or integrative Medicine	❍
8	Dosing & administration	❍
9	Others	❍

ASK ALL

C13a.

On a scale of 1 to 5 where 1 = ‘Extremely dissatisfied’ and 5 = ‘Extremely satisfied’, how satisfied are you with the current information sources for melanoma patients?

	Satisfaction with information sources	
1	Extremely Dissatisfied	❍
2		❍
3	Neutral	❍
4		❍

5	Extremely Satisfied	❍

ASK if C13a=1, 2

C13b.

You mentioned that you are dissatisfied with the current information sources for melanoma patients. Why are you dissatisfied with these information sources?

Select all that apply

Multi code possible

	Reasons for dissatisfaction	
1	Current Information sources not easily accessible	❍
2	Current Information sources not relevant	❍
3	Current Information sources hard to understand / not patient-friendly	❍
4	Other reasons	❍

SECTION D : MELANOMA AWARENESS , DIAGNOSIS & REFERRAL

ASK ALL

D1.

Were you aware of melanoma before being diagnosed with melanoma yourself?
Please select 1 option
Single code only

1	Yes	❑
2	No	❑

ASK if D1=1

D2.

What did you know about melanoma before being diagnosed with melanoma yourself?
Please be as specific as possible
Select all that apply
Multi code possible

	melanoma knowledge	
1	melanoma can be caused by exposure to UV rays	❑
2	melanoma can spread to other organs and can be deadly	❑
3	melanoma risk can be minimized by avoiding exposure to sun	❑
4	melanoma risk can be minimized by using sun screen	❑
6	Early detection and immediate treatment is critical for melanoma prognosis	❑
7	How a self-assessment of moles should be carried out	❑
7	Tanning beds are not healthier/safer than sitting in the sun	❑
8	Other topics	❑

ASK ALL

D4a.

Thinking about the circumstances under which you were diagnosed with melanoma, what triggered the first visit to the physician that eventually led to your diagnosis?
Select all that apply
Multi code possible

	melanoma diagnosis symptoms	
1	Sores that would not heal	❑
2	Pigment, redness or swelling that spread outside the border of a spot to the surrounding skin	❑
3	Itchiness, tenderness or pain	❑
4	Changes in texture, or scales, oozing or bleeding from an existing mole	❑

5	New or unusual growths on the skin	❍
6	Blurry vision or partial loss of sight, or dark spots on the iris	❍
7	No special trigger	❍
8	During routine skin check-up	❍
9	By coincidence	❍
10	Recommended by partner/friend/family member	❍
11	Disease awareness campaign	❍
11	Other	❍

ASK ALL

D4c.

What was the practicing specialty of the physician whom you reached out to first?

Please select 1 option

Single code only

	Practicing speciality of Initial Physician	
1	General Physician	❍
2	Dermatologist	❍
3	Oncologist	
4	Surgeon	
5	Other	❍

ASK ALL

D4d.

Did you continue with this initial physician through subsequent diagnosis and treatment stages for melanoma?

Please select 1 option

Single code only

1	Yes	❍
2	No	❍

ASK if D4d=1

D4e.

What additional roles did the initial physician play in your subsequent diagnosis and treatment for melanoma?

Select all that apply

Multi code possible

	Roles played by initial physician	
1	Made the melanoma diagnosis	❍
2	Referred me to specialist physician	❍
3	Consultant on treatment decision	❍

4	Knowledge support	❍
5	Psychological support	❍
6	Follow up management	❍
7	Side effect management	❍
8	Others	❍

ASK ALL

D6.

How long after an initial suspicion of melanoma did a skin specialist e.g. a dermatologist or a surgeon cut out the mole? *Please select 1 option*

Single code only

	Time from first Physician visit to Dermatologist /Specialist visit	
1	It was done the same appointment	❍
2	Less than 1 week	❍
3	Between 1 and 2 weeks	❍
4	Between 2 weeks and 4 weeks	❍
5	More than 4 weeks	❍
6	I didn't see a skin specialist after initial suspicion, my GP cut the mole out on the same appointment	❍
7	I didn't see a skin specialist after initial suspicion, my GP cut the mole out on a later appointment	❍

ASK ALL

D8.

How long did it take from taking biopsy to receive the diagnosis of melanoma?
Please select 1 option

Single code only

	Time to get biopsy results	
1	Less than 1 week	❍
2	Between 1 and 2 weeks	❍
3	Between 2 weeks and 4 weeks	❍
4	More than 4 weeks	❍
5	None of the above. I did not undergo a biopsy.	❍
6	I don't rembember	❍

ASK ALL

D10a.

How did your physician inform you about your melanoma diagnosis?

Please select 1 option

Single code only

	Communication Channel for melanoma Diagnosis	
1	Telephone	❍
2	E-mail	❍
3	Face to face conversation	❍
4	Instant messaging like WhatsApp	❍
5	Letter in the post	❍
6	I don't know it anymore	
7	Other	❍

ASK if D10a=1,3

D10c.

How long did this melanoma diagnosis conversation with the Physician last?

Please select 1 option

Single code only

	Length of melanoma diagnosis conversation	
1	less than 5 minutes	❍
2	5 to less than 15 minutes	❍
3	15 to less than 30 minutes	❍
4	30 to less than 60 minutes	❍
5	more than 60 minutes	❍
6	I did not have any conversation	❍
6	I don't remember anymore	❍

ASK ALL

D10d.

How satisfied were you with the way your melanoma diagnosis was communicated to you?

	Satisfaction with melanoma diagnosis communication	
1	Extremely dissatisfied	❍
2		❍
3	Neutral	❍
4		❍
5	Extremely satisfied	❍

ASK if D10d=1, 2

D10e.

You mentioned that you are dissatisfied with the way your melanoma diagnosis was communicated to you. What could have been improved to increase your satisfaction?

Select all that apply

Multi code possible

	Ways to improve satisfaction with melanoma Diagnosis communication	
1	Having a face to face interaction on melanoma diagnosis	❍
2	Longer first discussion about the melanoma diagnosis	❍
3	Show empathy in the discussion	❍
4	Show hope in the discussion	❍
5	Being clearer on how my future patient journey will look like	❍
6	Having more information / informational material on melanoma and treatment options	❍
7	Others	❍

ASK ALL

D11a.

At the time of your initial diagnosis, did the healthcare professional tell you about:

Select all that apply

Multi code possible

	Randomize	
1	Treatment options available	❍
2	Complementary therapies available (and some being paid by the insurance)	❍
3	Cost of treatment	❍
4	Mutational testing	❍
5	How to manage potential side effects of treatments	❍
6	How to tell family / friends	❍
7	Where to go for information	❍
8	Where to go for emotional/psychological support	❍
9	Where to go for financial support	❍
10	How to contact patient support groups	❍
11	Not sure / can't remember **(Exclusive option. Anchor)**	❍
12	None of the above **(EXCLUSIVE OPTION)**	❍

ASK ALL

D11b.

Overall, how useful would you say the information discussed after diagnosis was?

	Usefulness of information	
1	Not at all useful	❑
2		❑
3	Neutral	❑
4		❑
5	Very useful	❑

ASK ALL

D12.

What information sources for melanoma patients did you use to gain knowledge on melanoma –just after you were diagnosed with melanoma?
Which of these Information sources did you consider the most useful?

	information sources	Used D12a Multi code	Preferred D12b Single code
1	Physician	❑	❑
2	Patient brochures	❑	❑
3	Online Cancer patient forums	❑	❑
4	Friend	❑	❑
5	Internet	❑	❑
6	Patient group	❑	❑
7	Newspapers / Magazines	❑	❑
8	Television / Radio	❑	❑
9	Family	❑	❑
10	Others	❑	❑
11	None	❑	❑

ASK ALL

D13.

What psychological support systems for melanoma patients did you become aware of - just after you were diagnosed with melanoma?
Which of these support systems did you actually use?
And which support system did you find the most useful?

	Support systems	Aware of D13a Multi code	Used D13b Multi code	Most useful D13c Single code
1	Friends and Family	❑	❑	❑
2	Colleagues	❑	❑	❑
3	Religion / spiritual support	❑	❑	❑

4	Patient support groups	❍	❍	❍
5	Treating physician	❍	❍	❍
6	certified psychologist / psychooncologist	❍	❍	❍
7	melanoma / Skin Cancer Nurse	❍	❍	❍
8	Krebsliga Schweiz/Österreichische Krebshilfe	❍	❍	❍
9	Support group melanoma	❍	❍	❍
10	Others	❍	❍	❍
11	None	❍	❍	❍

ASK ALL

D14a.

When did you first talk to your family or close friends about your melanoma?

Please select 1 option

Single code only

	Time when family was informed	
1	When my melanoma symptoms appeared	❍
2	When the melanoma biopsy was conducted	❍
3	When my melanoma diagnosis was shared with me	❍
4	When my melanoma treatment started	❍
5	I didn't inform my family	❍
6	I prefer not to answer this question	❍

ASK if D14a=1,2,3,4

D14b.

What was your family's initial reaction to the news of your melanoma diagnosis?

	Inital reaction	
1	Confused	❍
2	Worried	❍
3	They offered their support	❍
4	They were looking for more information about melanoma	❍
5	Other reaction	❍
6	I prefer not to answer this question	❍

5. END

This is the end of the questionnaire. Thank you very much for your time and commitment in answering our questions.
